# First Characterization and Zoonotic Potential of *Cryptosporidium* spp. and *Giardia duodenalis* in Pigs in Hubei Province of China

**DOI:** 10.3389/fcimb.2022.949773

**Published:** 2022-07-11

**Authors:** Dongfang Li, Han Deng, Yaxin Zheng, Hongyan Zhang, Sen Wang, Lan He, Junlong Zhao

**Affiliations:** ^1^ State Key Laboratory of Agricultural Microbiology, College of Veterinary Medicine, Huazhong Agricultural University, Wuhan, China; ^2^ Key Laboratory of Preventive Veterinary Medicine in Hubei Province, Wuhan, China

**Keywords:** *Cryptosporidium*, *Giardia duodenalis*, pigs, infection rates, zoonotic

## Abstract

The zoonotic protozoa parasites *Cryptosporidium* spp. and *Giardia duodenalis* infect a wide range of hosts, including humans. Pigs are reservoir hosts for *Cryptosporidium* spp. and *G. duodenalis*, which can transmit cryptosporidiosis and giardiasis to humans and other animals. The current study sought to investigate the infection rates and species/genotypes of *Cryptosporidium* spp. and *G. duodenalis* in pigs in Hubei of China. The nested PCR and sequence analyses of the small ribosomal subunit RNA (*SSU* rRNA) gene for *Cryptosporidium* spp. and the β-giardin (bg) gene for *G. duodenalis* was utilized to screen for the infection of those parasites in a total of 826 fresh fecal samples. Both *Cryptosporidium* spp. and *G. duodenalis* infection rates were 0.97% (8/826). Of the genotyped positive isolates, 6/8 (75%) were *C. scrofarum* and 2/8 (25%) were *C. suis*, while two zoonotic species *G. duodenalis* assemblage E and assemblage A were also detected in 7/8 (87.5%) isolates and 1/8 (12.5%) isolates, respectively. The findings suggest that both of those parasites in pig in intensive farms of Hubei province, China, pose a potential public health risk.

## Introduction

*Cryptosporidium* spp. and *Giardia duodenalis* were found worldwide, infecting a wide variety of vertebrate hosts and causing self-limiting diarrhea and other clinical signs in humans and livestock, especially in immunodeficient or immunocompromised individuals (Ryan et al., 2014; Ryan et al., 2019). Each genus comprises a complex of species and genotypes, some of which are zoonotic and some specific to particular hosts (Feng et al., 2018; Naguib et al., 2021). People can be infected by directly or indirectly ingesting infective *Cryptosporidium* oocysts and *Giardia* cysts, *via* contaminated water, food and pasture (Thompson and Ash, 2016). In fact, in the 24 foodborne parasites species listed by Food and Agriculture Organization of the United Nations (FAO), *Cryptosporidium* spp. and *G. duodenalis* were ranked fifth and 11th, respectively.

To date, at least 40 *Cryptosporidium* species and 100 genotypes, as well as nine *G. duodenalis* assemblages have been identified (Ryan et al., 2021). Based on molecular identification, *C. parvum* and *C. hominis* are the most common causes of human cryptosporidiosis. *C. meleagridis*, *C. ubiquitum*, *C. cuniculus*, *C. felis*, *C. canis*, *C. viatorum*, and *C. muris* are some of the other human-pathogenic Cryptosporidium spp (Feng et al., 2018). *Giardia duodenalis* is a species complex consisting of nine genetically distinct assemblages (A-H), and the common assemblages A and B usually infect humans and other mammals, whereas assemblages C-H only infect specific host (Caccio and Ryan, 2008). *Cryptosporidium* and *Giardia* have been reported in pigs from almost all countries or regions of the world. Six *Cryptosporidium* species have been isolated in pigs: *C. suis*, *C. parvum*, *C. muris*, *C. andersoni*, *C. scrofarum* (formerly *Cryptosporidium* pig genotype II), and *C. tyzzeri* (formerly *Cryptosporidium* mouse genotype I) (Zheng et al., 2019). The main *Cryptosporidium* species identified in pigs worldwide are *C. suis* and *C. scrofarum* (Feng et al., 2018). However, these two pig-adapted *Cryptosporidium* species have been repeatedly identified in fecal samples from human, which indicated zoonotic potential (Leoni et al., 2006; Chen et al., 2011; Bodager et al., 2015). *G. duodenalis* assemblage A was commonly reported in humans and has been identified in pig (Langkjaer et al., 2007; Caccio et al., 2008; Armson et al., 2009). Swine dung may pollute the environment through water or other ways, and pasture runoff can introduce enormous quantities of *Cryptosporidium* oocysts and *Giardia* spores into streams and rivers. Pigs were infected with zoonotic species and genotypes of *Cryptosporidium* and *G. duodenalis*, suggesting that they might be sources of infection for nearby residents.

In China, the pig sector is the most important in animal production, and the *Cryptosporidium* and *G. duodenalis* infection in pigs has been recorded in most regions of China (Chen et al., 2011; Zhang et al., 2013; Lin et al., 2015; Wang et al., 2017a; Liu et al., 2019; Zheng et al., 2019). However, no studies have investigated *Cryptosporidium* spp. and *G. duodenalis* in pigs in Hubei of China. Therefore, the focus of this study was to determine the prevalence and genotypes of *Cryptosporidium* and *G. duodenalis* in domestic pigs of various age groups in Hubei, central region of China, and elucidate the role they play in human and animals health under the One Health concept.

## Materials and Methods

### Ethical Approval

The Research Ethics Committee of Huazhong Agricultural University reviewed and approved our study. Prior to sample collection, we obtained permission from the head of the animal farms.

### Sampling

**Figure 1:**
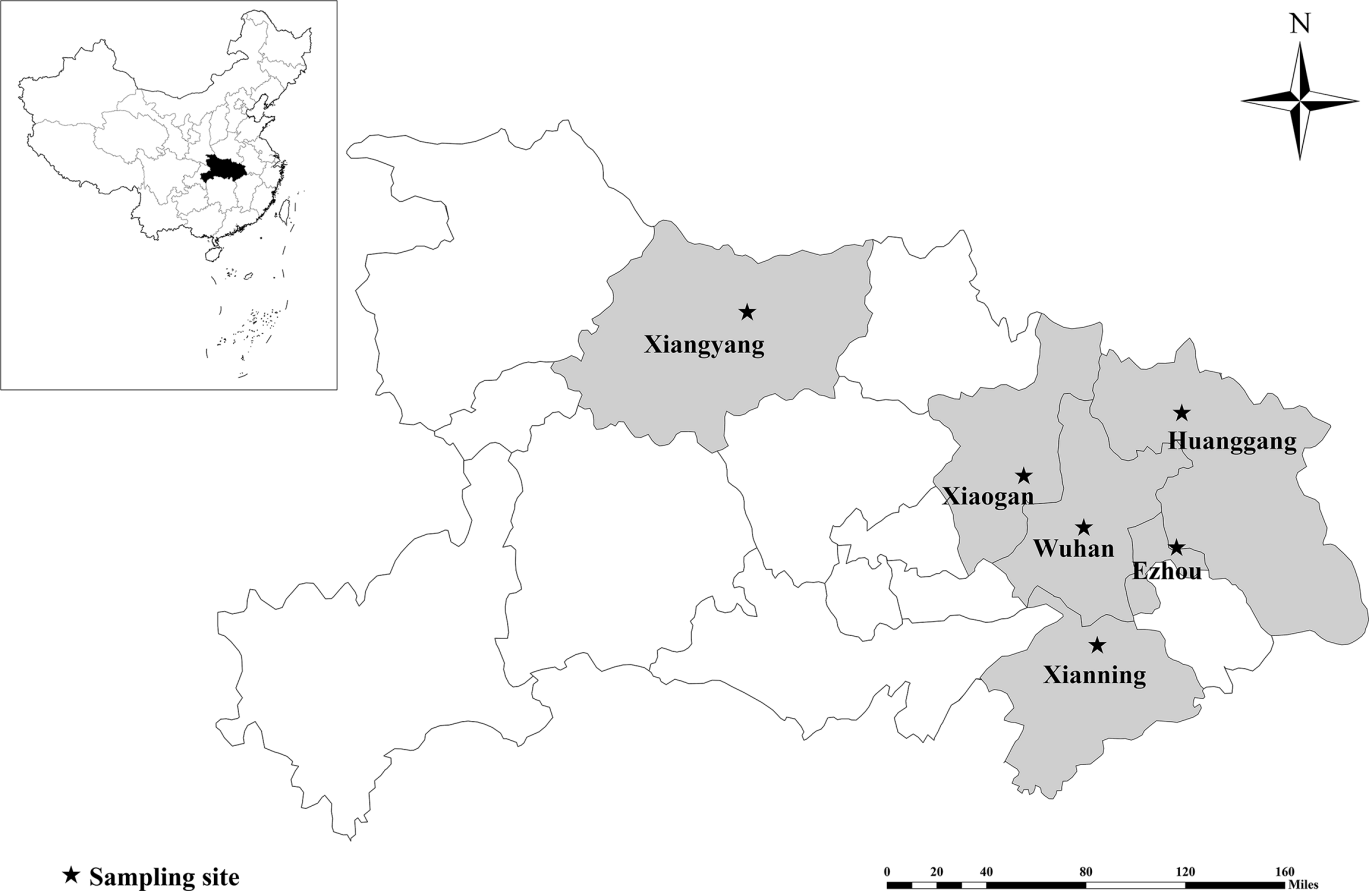
Sampling sites in pigs in Hubei Province, China. Sampling locations are marked in dark colors.

Between September and December of 2019, 826 fresh fecal specimens of pigs were obtained from nine intensive pig farms located at Xianning, Xiaogan, Wuhan, Huanggang, Ezhou and Xiangyang areas in Hubei (**Figure 1**). The fecal samples were collected from different age groups, including 153 from pre-weaned piglets (<20 days), 231 from post-weaned piglets (21-70 days), 80 from fattening pigs (71-180 days), 362 from sows and herd boar (>180 days). A veterinarian assisted in the collection of fecal specimens from the rectum or the internal component of a stool specimen found on the ground. All specimens (weighing between 5 and 30 g) were gathered with sterile disposable gloves, labeled with the date, age, and farm, and transported to laboratory in cold containers with ice packs. When fecal specimens were taken froom the pigs, no clear clinical symptoms was observed.

### DNA Extraction and PCR Amplification

The TIANamp Stool DNA Kit (TIANGEN BIOTECH(BEIJING) CO., LTD, Beijing, China) was used to extract total genomic DNA from about 200 mg of each fecal material according to the manufacturer’s instructions, each DNA specimen was given 50 μL of elution buffer and stored at −20°C until PCR amplification.

As previously stated, *Cryptosporidium* and *Giardia* was screened *via* using nested PCR amplification of the small ribosomal subunit RNA (*SSU* rRNA) gene and β-giardin (bg) gene loci, respectively (Xiao et al., 1999; Lalle et al., 2005). For *Cryptosporidium* and *G. duodenalis*, rTaq and Extaq (Takara Bio Inc., Dalian, China) were used in a 25 μL PCR amplification system, containing 3.5 μL 10 ×PCR Buffer, 2 μL dNTP Mix (2.5 mmol/L), 0.5 μL of forward and reverse primers (25 μmol/L), 15.3 μL deionized water, 1μL rTaq (1.25 U) or ExTaq (1.25 U), 2 μL genomic DNA for the primary PCR template, and 2 μL primary amplification product used for the secondary PCR template. Positive controls (Cattle-derived *C. parvum* and *G. duodenalis* assemblage E DNA) and negative controls (distilled water) were included in each PCR assay. Each sample was analyzed by PCR with two technical replicates for each gene locus. All secondary PCR products were electrophoresed in a 1.5% agarose gel, then visualized *via* GelRed staining and a UV transilluminator (ProteinSimple Inc., State of California, USA).

### Nucleotide Sequencing and Analysis

The target gene’s positive PCR amplicons were direct sequenced bidirectionally using secondary primers of sanger sequencing in TSINGKE Biological Technology (Wuhan, China). To determine the species and subtypes of *Cryptosporidium* and *G. duodenalis*, all nucleotide sequences were run through the Basic Local Alignment Search tool (BLAST) and compared to *Cryptosporidium* and *Giardia duodenalis* reference sequences downloaded from the National Center for Biotechnology Information (https://www.ncbi.nlm.nih.gov/) using ClustalX 2.1 (2010-11-17) (http://www.clustal.org/).

### Statistical Analysis and Nucleotide Sequence Accession Numbers

The χ2 test was utilized to compare the *Cryptosporidium* or *Giardia* prevalence from pigs in different sample region or age group using SPSS 22.0. Statistical significance was established at p<0.05. The representative *Cryptosporidium* and *G. duodenalis* nucleotide sequences identified in the pigs were submitted to GenBank at the National Center for Biotechnology Information under accession numbers: ON149804-ON149811 for *Cryptosporidium* and ON168862-ON168869 for *Giardia*.

## Results

### Prevalence of *Cryptosporidium* spp. and *Giardia duodenalis*

**Figure 2:**
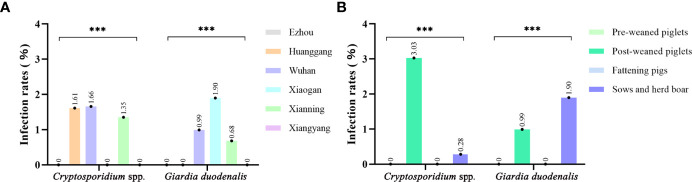
Occurrence of *Cryptosporidium* spp. and *Giardia duodenalis* in different sample site **(A)** and age group **(B)** of pigs from Hubei Province, China. ***p<0.001.

Of the 826 fecal specimens collected from pigs, the infection of *Cryptosporidium* 0.97% (8/826), and *G. duodenalis* were also 0.97% (8/826). Of the 6 sampled regions in this study, only 3 regions were positive for *Cryptosporidium*, and the prevalence of *Cryptosporidium* in pigs in different regions was between 0 and 1.66% (**Table 1**). Among them, Wuhan City is the highest (0.66%, 5/302), followed by Huanggang (1.61%, 1/62), Xianning (1.35%, 2/148), and no *Cryptosporidium* were detected in pig farms from Xiaogan, Ezhou and Xiangyang. In addition, in four pigs age group, the highest infection rate was in nursery pigs (3.03%, 7/231), followed by gilts (0.28%, 1/362), and no *Cryptosporidium* infection was found in the other age pig herds (**Table 2**). Significantly difference of *Cryptosporidium* infection rates was observed among sampled regions (χ2 = 4140, p < 0.001) and pigs aged (χ2 = 4130, p < 0.001) (**Figure 2**).

**Table 1:** Prevalence and genetic characterizations of *Cryptosporidium* spp. and *Giardia duodenalis* arranged by sampled location in pigs in Hubei of China.

Location	Number tested	*Cryptosporidium* spp.		*Giardia duodenalis*	
		Infection rates	Species	Infection rates	Species
Ezhou	80	0		0	–
Huanggang	62	1 (1.61%)	*C. scrofarum* (1)	0	–
Wuhan	302	5 (1.66%)	*C. scrofarum* (3), *C. suis* (2)	3 (0.99%)	E (2), A (1)
Xiaogan	210	0		4 (1.90%)	E (4)
Xianning	148	2 (1.35%)	*C. scrofarum* (2)	1 (0.68%)	E (1)
Xiangyang	24	0		0	–
Total	826	8 (0.97%)	*C. scrofarum* (6), *C. suis* (2)	8 (0.97%)	E (7), A (1)

**Table 2:** Prevalence and genetic characterizations of *Cryptosporidium* spp. and *Giardia duodenalis* arranged by age group in pigs in Hubei of China.

Age	Number tested	*Cryptosporidium* spp.		*Giardia duodenalis*	
		Infection rates	Species	Infection rates	Species
Pre-weaned piglets	153	0	–	0	–
Post-weaned piglets	231	7 (3.03%)	*C. scrofarum* (5), *C. suis* (2)	3 (1.30%)	E (2), A (1)
Fattening pigs	80	0	–	0	–
Sows and herd boar	362	1 (0.28%)	*C. scrofarum* (1)	5 (1.38%)	E (5)
Total	826	8 (0.97%)	*C. scrofarum* (6), *C. suis* (2)	8 (0.97%)	E (7), A (1)

The prevalence of *G. duodenalis* in pigs in different regions ranged from 0 to 1.90%. Among them, Xiaogan has the highest infection rate (1.90%, 4/210), followed by Wuhan City (0.99%, 3/102), Xianning (0.68%, 1/148), while no *G. duodenalis* were detected in the pig farms from Huanggang, Ezhou and Xiangyang. Among the pigs of all ages, the highest infection rate was in sows and herd boar (1.38%, 5/362), followed by post-weaned pigs (1.30%, 3/231), and no *G. duodenalis* infection was found in the other age group. Significantly difference of *G. duodenalis* infection rates was observed among sampled regions (χ2 = 2481, p < 0.001) and pigs age group (χ2 = 2478, p < 0.001).

### Molecular Characterization of *Cryptosporidium* spp. and *Giardia duodenalis*

Based on sequence analysis, six and two of eight *Cryptosporidium* positive samples were identified as *C. scrofarum* and *C. suis*, respectively. Among the three positive city, *C. scrofarum* was identified in Wuhan (n=3), Xianning (n=2) and Huanggang (n=1), while *C. suis* was only identified in Wuhan (n=2). *C. scrofarum* were both identified in the two positive age groups, with 5 in post-weaned pigs and 2 in sows and herd boar, while only one *C. suis* positive samples were identified in post-weaned pigs. Of the eight *G. duodenalis* positive samples, sequence analysis revealed seven assemblage E and one assemblage A based on BG. Assemblage E was identified in three positive regions, including Wuhan (n=2), Xiaogan (n=4) and Xianning (n=1), while assemblage A was identified in Wuhan (n=1). In addition, assemblage E (n=2) and assemblage A (n=1) were found in post-weaned piglets, and only assemblage E (n=4) was found in Snows and herd boar.

## Discussion

The previously reported prevalence of *Cryptosporidium* and *G. duodenalis* in pigs from most area of China, and have been reported in most country of the world. The infection rates of *Cryptosporidium* in pigs were much lower than those reported in Asian countries such as Japan (32.6%) (Yui et al., 2014), Thailand (20.8%) (Thathaisong et al., 2020), Vietnam (14.5%) (Nguyen et al., 2013), and far lower than the total infection rate in China (12.2%), and also lower than those reported in infection rates reported in other provincial areas such as Yunnan (23.0%, 46/200), Zhejiang (14.5%, 18/124), Fujian (11.9%, 16/135), Guangdong (8.3%, 34/217), Jiangxi (4.0%, 41/1036), Shaanxi (3.3%, 44/1337), Henan (3.1%, 28/897), and Heilongjiang (1.6%, 9/568) (Zhang et al., 2013; Lin et al., 2015; Zou et al., 2017; Wang et al., 2018; Wang et al., 2021; Wang et al., 2022). Nested PCR amplification based on the BG gene of *G. duodenalis* showed that a total of 8 positive samples were amplified with an overall infection rate of 0.97% (8/826), which is much lower than the infection rates previously reported in Denmark (14.0%, 120/857) (Petersen et al., 2015), Korea (14.8%, 110/745) (Lee et al., 2020), and also lower than the previous infection rates reported in other regions of China such as Shanghai (26.9%, 25/93), Zhejiang (10.5%, 13/124), Shaanxi (8.0%, 45/560), Yunnan (5.3%, 21/396; 2.5%, 5/200), Taiwan (4.3%, 6/141), Guangdong (4.2%, 3/72), Xinjiang (2.6%, 21/801), and Henan (1.7%, 15/897) (Wang et al., 2017a; Wang et al., 2018; Jing et al., 2019; Liu et al., 2019; Lam et al., 2021; Zou et al., 2021). There are many reasons for such a large variation in infection rates, which may be related to the number, time and location of sampling, the size and environment of pig farms, animal population density, detection methods and other geographical factors such as temperature and humidity, precipitation and climate (Wang et al., 2021). The main reason for the generally lower infection rates in this study than those previously reported in other regions of China may be that the sampling time of this study was right after the outbreak of African swine fever in China, and most of the pig farms were in the resumption phase as well as in the phase of strict prevention and control of African swine fever, and all large-scale pig farms around the country had greatly improved their biosecurity level, and the frequency and degree of disinfection of the environment in pig farms had been enhanced compared to the previous ones.

Two *Cryptosporidium* species, *C. scrofarum* and *C. suis*, were identified in this study, and the results are consistent with those reported in other provinces of China, and these two *Cryptosporidium* species were frequently identified in pigs worldwide, and both are human-animal species. In addition, *C. parvum*, which mainly infects humans, has been reported several times in foreign swine surveys, but is rarely reported in China (Ryan et al., 2014). *C. suis* mainly infects pigs, have also been isolated from water source and tap water samples in Shanghai, China (Feng et al., 2011). A total of two *G. duodenalis* assemblages were identified, with seven samples being assemblage E and one being assemblage A. This result was similar to that reported in Shaanxi Province (Wang et al., 2017a), assemblage E was the dominant genotype, and like the results reported for *G. duodenalis* of porcine origin in other regions of China (Liu et al., 2019). In contrast, assemblage A is capable of infecting animals such as humans and domestic animals, and was identified in this study and in swine herds in Shaanxi, Shanghai, and in children with diarrhea in Wuhan, indicating that assemblage A is currently prevalent among both humans and swine (Wang et al., 2017a; Wang et al., 2017b; Liu et al., 2019).

Cases of *C. scrofarum* and *C. suis* infections have been reported in immunocompetent diarrhea patients and HIV-positive patients in recent years, suggesting that these two pig-adapted *Cryptosporidium* species may be zoonotic (Xiao et al., 2006; Bodager et al., 2015). *C. suis* was first identified in a 24-year-old HIV patient in Peru and China (Xiao et al., 2002; Cama et al., 2007; Wang et al., 2013). *C. suis* has also been identified in patients with digestive diseases in the UK and Madagascar (Leoni et al., 2006; Bodager et al., 2015). Only one case of human infection with *C. scrofarum* has been reported, which occurred in the Czech Republic (Kvac et al., 2009). Cryptosporidiosis in pigs should receive more attention because it is not only a veterinary problem but may be important for public health. More important, *C. suis* has been detected in drinking water source water (Feng et al., 2011; Xiao et al., 2012; Hu et al., 2014). *G. duodenalis* assemblage A and E were frequently found in pigs around the world (Jing et al., 2019; Zou et al., 2021). In addition, assemblage A is one of the main assemblages of infected people, and assemblage E was identified in humans in Australia (Zahedi et al., 2017; Cai et al., 2021). All these reports indicate that pig populations play an important role in the transmission of *Cryptosporidium* and *G. duodenalis* are of great public health importance. Therefore, there is a need to develop better farm management systems to reduce environmental contamination by zoonotic diseases and prevent the occurrence of zoonotic transmission caused by *Cryptosporidium* and *G. duodenalis*. The prevention and control of those parasites in large-scale pig farms requires strengthening the nutritional management of the herd, improving the immunity of the herd, selecting drugs with anti-oocyst activity to enhance the disinfection of environmental hygiene as well as taking effective measures to control insects such as flies, enhancing the cleaning of manure and keeping the pen dry.

## Conclusion

This study reported the prevalence of *Cryptosporidium* and *G. duodenalis* infection in healthy pigs. Due to the zoonotic nature of *Cryptosporidium* spp. and *G. duodenalis*, adequate cleaning and sanitary activities in pig farms should be used to prevent probale transfer of infective parasites to humans and other animals.

## Data Availability Statement

The datasets presented in this study can be found in online repositories. The names of the repository/repositories and accession number(s) can be found in the article/supplementary material.

## Author Contributions

JZ and LH conceived and designed this study. DL, HD, YZ and HZ conducted the laboratory work. DL drafted the manuscript. JZ, LH and SW critically revised the manuscript. All authors have read and approved the final manuscript.

## Funding

This work was supported by the National Key Research and Development Program Intergovernmental International Cooperation Project of China (Grant No. SQ2021YFE012313), and the Fundamental Research Funds for the Central Universities in China (Project 2662020DKPY016).

## Conflict of Interest

The authors declare that the research was conducted in the absence of any commercial or financial relationships that could be construed as a potential conflict of interest.

## Publisher’s Note

All claims expressed in this article are solely those of the authors and do not necessarily represent those of their affiliated organizations, or those of the publisher, the editors and the reviewers. Any product that may be evaluated in this article, or claim that may be made by its manufacturer, is not guaranteed or endorsed by the publisher.
